# Robust multivariate nonparametric tests for detection of two-sample location shift in clinical trials

**DOI:** 10.1371/journal.pone.0195894

**Published:** 2018-04-19

**Authors:** Xuejun Jiang, Xu Guo, Ning Zhang, Bo Wang, Bo Zhang

**Affiliations:** 1 Department of Mathematics, Southern University of Science and Technology, Shenzhen, Guangzhou, P.R. China; 2 Department of Mathematical Statistics, School of Statistics, Beijing Normal University, Beijing, P.R. China; 3 Department of Health Promotion and Policy, School of Public Health and Health Sciences, University of Massachusetts, Amherst, Massachusetts, United States of America; 4 Meyers Primary Care Institute, A Joint Endeavor of University of Massachusetts Medical School, Reliant Medical Group, and Fallon Health, Worcester, Massachusetts, United States of America; 5 Department of Quantitative Health Sciences, University of Massachusetts Medical School, Worcester, Massachusetts, United States of America; University of the Chinese Academy of Sciences, CHINA

## Abstract

This article presents and investigates performance of a series of robust multivariate nonparametric tests for detection of location shift between two multivariate samples in randomized controlled trials. The tests are built upon robust estimators of distribution locations (medians, Hodges-Lehmann estimators, and an extended *U* statistic) with both unscaled and scaled versions. The nonparametric tests are robust to outliers and do not assume that the two samples are drawn from multivariate normal distributions. Bootstrap and permutation approaches are introduced for determining the *p*-values of the proposed test statistics. Simulation studies are conducted and numerical results are reported to examine performance of the proposed statistical tests. The numerical results demonstrate that the robust multivariate nonparametric tests constructed from the Hodges-Lehmann estimators are more efficient than those based on medians and the extended *U* statistic. The permutation approach can provide a more stringent control of Type I error and is generally more powerful than the bootstrap procedure. The proposed robust nonparametric tests are applied to detect multivariate distributional difference between the intervention and control groups in the Thai Healthy Choices study and examine the intervention effect of a four-session motivational interviewing-based intervention developed in the study to reduce risk behaviors among youth living with HIV.

## Introduction

In randomized controlled trials, effectiveness (or efficacy) of a treatment effect is constantly characterized by the difference between distributional locations of a treatment group and its control group. Hypothesis testing is the primary statistical inference approach in examining treatment effects in clinical trials, when it is conducted to detect whether there exists any difference between distributional locations of the treatment group and the control group. When the primary endpoint is one-dimensional and normally distributed for both study groups, the two-sample *t* test is the standard tool. Yet, the two-sample *t* test may not be valid when normality assumption is violated. The two-sample *t* test is not robust to outliers and heavy-tail distributions. A number of robust nonparametric tests have been developed in the literature as a complement of the two-sample *t* test. The classic Wilcoxon-Mann-Whitney test [1] that used the rank sum is a nonparametric counterpart of the two-sample *t* test. Yuen [2] and Keselman et al. [3] recommended to construct the tests using trimmed means. Recently, Fried and Dehling [4] proposed a series of robust nonparametric tests for detecting univariate two-sample location difference. These tests were constructed based upon unscaled and properly scaled robust location estimators of distributions, including medians and Hodges-Lehmann estimators. The numerical studies reported by Fried and Dehling [4] showed that the test statistics were robust to outliers and non-normality and efficient in detecting univariate two-sample location shift. Mathur [5] proposed a strictly nonparametric bivariate test constructed from an extended *U* statistic and concluded that the test statistic did not depend on the covariance structure of the underlying population and was more powerful than the existing tests.

In randomized controlled trials, effectiveness of a treatment effect can be defined by not a single or two, but multiple primary endpoints, and significance of the treatment effect is then determined by multivariate location shift between the two multivariate distributions of the treatment and control groups. In these clinical trials, multivariate hypothesis testing procedures are needed to detect a potential location shift between two samples that are drawn with a multivariate primary endpoint. The conventional univariate two-sample *t* test was extended to the multivariate setting by Hotelling [6] and the proposed test statistic was denominated Hotelling’s *T*^2^ statistic. The Hotelling’s *T*^2^ test inherits the limitations of univariate two-sample *t* test, because it is still not robust to multivariate outliers and not valid when the multivariate normality assumption is violated. This motivated the development of multivariate two-sample location tests. Hettmansperger and Oja [7] developed a multivariate sign test for detecting location deviation among multiple multivariate samples. Hettmansperger et al. [8] introduced affine invariant analogues of the two-sample Mann-Whitney-Wilcoxon rank sum test. Neuhaus and Zhu [9] proposed multivariate distribution-free permutation test statistics that were built upon projected univariate versions of multivariate data. Henze et al. [10] introduced a class of consistent tests, in which the test statistic is a weighted integral of the squared modulus of the difference of the empirical characteristic functions of one multivariate sample and another multivariate sample plus a location shift.

In this article, we extend the robust nonparametric test statistics proposed by Fried and Dehling [4] and Mathur [5] to the multivariate setting. A series of robust multivariate nonparametric tests are proposed using the component-wise medians, Hodges-Lehmann location estimators, and an extended *U* statistic. Univariate test statistics for detecting multivariate two-sample location shift are constructed for the robust multivariate nonparametric tests as (i) unscaled maximum of the component-wise medians or the Hodges-Lehmann estimators, (ii) scaled maximum of the component-wise medians or the Hodges-Lehmann estimators, (iii) maximum of the scaled component-wise medians or the Hodges-Lehmann estimators, or (iv) the extended *U* statistic. A bootstrap approach and a permutation approach are introduced for determining the *p*-values of the proposed test statistics. We conduct simulation studies to examine performance of the proposed robust nonparametric test statistics in detecting multivariate two-sample location shift. The numerical results given by the bootstrap procedure demonstrate that the proposed robust multivariate nonparametric tests constructed from the Hodges-Lehmann estimators are more efficient than those based on medians and the extended *U* statistic. The permutation approach can provide a more stringent control of Type I error and is more powerful than the bootstrap procedure. To demonstrate the use of these proposed robust multivariate nonparametric tests, the proposed hypothesis tests are applied to detecting the intervention effect of the Thai Healthy Choices study [11], a study that promotes a four-session motivational interviewing-based intervention to reduce risk behaviors among youth living with HIV (the Thai Healthy Choices study was designed jointly by Wayne State University and the Thai Red Cross AIDS Research Center, and implemented in Bangkok, Thailand).

The scientific contribution of this article is multifold. First, a series of new robust nonparametric test statistics are proposed for detecting location shift between two multivariate samples collected from the treatment and control groups, respectively, in clinical trials. Both a bootstrap approach and a permutation approach are introduced to implement the proposed tests for seeking corresponding *p*-values. These provide the practitioners a variety of choices with two distinct implementation approaches to test on treatment effects when the normality assumption for the samples is violated. Second, comprehensive numerical studies are conducted and the results show explicit benefits from using the proposed tests over the Hotelling’s *T*^2^ and the extended *U* tests in term of controlling Type I error and boosting statistical power. Third, the article presents a representative example, the Thai Healthy Choices study, and shows how the proposed robust nonparametric hypothesis testing procedures can be implemented to test on the treatment or intervention effect in a clinical trial.

## Tests on two-sample location shift

Consider two random samples {*X*_1_, ⋯, *X*_*m*_} and {*Y*_1_, ⋯, *Y*_*n*_} with *p*-dimensional independent multivariate observations $X_{i}\in R^{p}$, *i* = 1, ⋯, *m*, and $Y_{j}\in R^{p}$, *j* = 1, ⋯, *n*, for which

$$\begin{array}{c} X_{1},\cdots ,X_{m}\overset{i.i.d}{∼}F\left(x\right) \end{array}$$

and

$$\begin{array}{c} Y_{1},\cdots ,Y_{n}\overset{i.i.d}{∼}G\left(x\right). \end{array}$$

The null hypothesis of equality of *F*(*x*) and *G*(*x*) and its alternative hypothesis that there is a location shift in the two multivariate distributions are

$$\begin{array}{c} H_{0}:\;\;F\left(x\right)=G\left(x\right)\;\text{versus}\;H_{1}:\;\;F\left(x\right)=G\left(x+\Delta \right). \end{array}$$ (1)

A natural idea to test the above hypotheses is to compare location estimators of the two distributions. Sample means $\bar{X}$ and $\bar{Y}$ can be used to fulfil this mission, which leads to the prominent Hotelling’s *T*^2^ test. However, the Hotelling’s *T*^2^ test is constructed under the multivariate normal distributions, and therefore perform poorly when there are outliers or the underlying true distributions of the two samples do not follow multivariate normal distributions.

### Tests based on unscaled median difference and Hodges-Lehmann estimators

Here, we propose a series of robust nonparametric test statistics based on robust estimators for distribution locations as competitors of the Hotelling’s *T*^2^ test statistics. A general approach to construct such nonparametric tests is to estimate the location difference Δ and then reject the null hypothesis *H*_0_ if Δ is far from zero. As usual, we can replace difference of sample means with difference between two sample medians:

$$\begin{array}{c} {\hat{\Delta }}_{1}=\text{med}\left\{Y_{1},\cdots ,Y_{n}\right\}-\text{med}\left\{X_{1},\cdots ,X_{m}\right\}. \end{array}$$ (2)

In (2), med{*X*_1_, ⋯, *X*_*m*_} and med{*Y*_1_, ⋯, *Y*_*n*_} are the *p*-dimensional median vectors of the two samples. The median vector of a sample is defined as the vector of component-wise medians. That is, the *k*th component of med{*X*_1_, ⋯, *X*_*m*_} is the median of *X*_1*k*_, ⋯, *X*_*mk*_, where *X*_*ik*_ is the *k*th component of *p*-dimensional observation *X*_*i*_ for *i* = 1, ⋯, *m*, and the *k*th component of med{*Y*_1_, ⋯, *Y*_*n*_} is the median of *Y*_1*k*_, ⋯, *Y*_*nk*_, where *Y*_*jk*_ is the *k*th component of *p*-dimensional observation *Y*_*j*_ for *j* = 1, ⋯, *n*. In practice, however, ${\hat{\Delta }}_{1}$ cannot be directly used as a test statistic for the pair of hypotheses in (1), since ${\hat{\Delta }}_{1}$ is a *p*-dimensional vector and is not a scalar. Therefore, the following maximum of absolute values of the *p* medians within ${\hat{\Delta }}_{1}$ can be considered:

$$\begin{array}{c} {\hat{\Delta }}_{1}^{\text{max}}=\max\{|{\hat{\Delta }}_{11}\left|,\right|{\hat{\Delta }}_{12}\left|,\cdots ,\right|{\hat{\Delta }}_{1p}\left|\right\}, \end{array}$$

where ${\hat{\Delta }}_{1k}$ is the *k*th component of ${\hat{\Delta }}_{1}$ for *k* = 1, ⋯, *p*. Under the null hypothesis ${\hat{\Delta }}_{1}^{\text{max}}$ should be close to zero, whereas under alternative hypothesis it deviates from zero.

Noted that, although the direct sample medians med{*X*_1_, ⋯, *X*_*m*_} and med{*Y*_1_, ⋯, *Y*_*n*_} in (2) are robust estimators for the locations of two samples, these medians are not very efficient as each of them exploits little information in the sample data. To balance efficiency against robustness, two types of Hodges-Lehmann estimators were developed [12, 13]. Multivariate analogs of the univariate Hodges-Lehmann estimators are

$$\begin{array}{c} {\hat{\Delta }}_{2}=\text{med}\left\{Y_{j}-X_{i};i=1,\cdots ,m,j=1,\cdots ,n\right\} \end{array}$$

and

$$\begin{array}{c} {\hat{\Delta }}_{3}=\frac{\text{med}\{Y_{i}+Y_{j};1\leq i<j\leq n\}}{2}-\frac{\text{med}\{X_{i}+X_{j};1\leq i<j\leq m\}}{2}, \end{array}$$

where the *p*-dimensional multivariate median vectors are likewise defined as in (2). The test statistics using the multivariate Hodges-Lehmann estimators to detect location shift between two multivariate samples are then proposed as the absolute component-wise maximum of ${\hat{\Delta }}_{2}$ and ${\hat{\Delta }}_{2}$:

$$\begin{array}{c} {\hat{\Delta }}_{2}^{\text{max}}=\max\{|{\hat{\Delta }}_{21}\left|,\right|{\hat{\Delta }}_{22}\left|,\cdots ,\right|{\hat{\Delta }}_{2p}\left|\right\} \end{array}$$

and

$$\begin{array}{c} {\hat{\Delta }}_{3}^{\text{max}}=\max\{|{\hat{\Delta }}_{31}\left|,\right|{\hat{\Delta }}_{32}\left|,\cdots ,\right|{\hat{\Delta }}_{3p}\left|\right\}. \end{array}$$

where ${\hat{\Delta }}_{2k}$ and ${\hat{\Delta }}_{3k}$ are the *k*th component of ${\hat{\Delta }}_{2}$ and ${\hat{\Delta }}_{3}$, respectively, for *k* = 1, ⋯, *p*.

### Tests based on scaled median difference and Hodges-Lehmann estimators

Because ${\hat{\Delta }}_{1}^{\text{max}}$, ${\hat{\Delta }}_{2}^{\text{max}}$ and ${\hat{\Delta }}_{3}^{\text{max}}$ only measure the component-wise maximum variability between the two samples, a scaled version of each is required to construct a more robust nonparametric test statistic. To this end, a related measure of the variability within the two samples are needed for the procedure of standardization. For ${\hat{\Delta }}_{1}^{\text{max}}$, the following *p*-dimensional median vector is the measure of component-wise differences between the two samples:

$$\begin{array}{c} S_{1}=2\;\text{med}\left\{Z_{i};i=1,\cdots ,m+n\right\}, \end{array}$$

where ${\left(Z_{1},\cdots ,Z_{m+n}\right)}^{\prime }={\left(X_{1}-\tilde{X},\cdots ,X_{m}-\tilde{X},Y_{1}-\tilde{Y},\cdots ,Y_{n}-\tilde{Y}\right)}^{\prime }$ is the joint median-corrected sample and $\tilde{X}=\text{med}\left\{X_{1},\cdots ,X_{m}\right\}$ and $\tilde{Y}=\text{med}\left\{Y_{1},\cdots ,X_{n}\right\}$. Then, the absolute component-wise maximum of *S*_1_ is defined as

$$\begin{array}{c} S_{1}^{\text{max}}=2\max\{\text{med}\{|Z_{i}|;i=1,\cdots ,m+n\}\}, \end{array}$$

and the standardized version of ${\hat{\Delta }}_{1}^{\text{max}}$ can be formulated as

$$\begin{array}{c} T_{1}={\hat{\Delta }}_{1}^{\text{max}}/S_{1}^{\text{max}}. \end{array}$$

For ${\hat{\Delta }}_{2}^{\text{max}}$ and ${\hat{\Delta }}_{3}^{\text{max}}$, the following *p*-dimensional median vectors of the absolute set of differences in the samples and within the joint median-corrected sample can be taken as the measure of component-wise differences within the two samples:

$$\begin{array}{c} S_{2}=\text{med}\{|X_{i}-X_{j}\left|,1\leq i<j\leq m;\right|Y_{i}-Y_{j}\left|,1\leq i<j\leq n\right\} \end{array}$$

and

$$\begin{array}{c} S_{3}=\text{med}\{|Z_{i}-Z_{j}\left|;1\leq i<j\leq m+n\right\}. \end{array}$$

Then, the absolute component-wise maximum of them are defined as

$$\begin{array}{c} S_{2}^{\text{max}}=\max\{\text{med}\{|X_{i}-X_{j}\left|,1\leq i<j\leq m;\right|Y_{i}-Y_{j}\left|,1\leq i<j\leq n\}\right\} \end{array}$$

and

$$\begin{array}{c} S_{3}^{\text{max}}=\max\{\text{med}\{|Z_{i}-Z_{j}|,1\leq i<j\leq m+n\}\}, \end{array}$$

respectively. The scaled versions of ${\hat{\Delta }}_{2}^{\text{max}}$ and ${\hat{\Delta }}_{3}^{\text{max}}$ are consequently constructed as

$$\begin{array}{c} T_{2}={\hat{\Delta }}_{2}^{\text{max}}/S_{2}^{\text{max}}\;\;\text{and}\;\;T_{3}={\hat{\Delta }}_{2}^{\text{max}}/S_{3}^{\text{max}}, \end{array}$$

and

$$\begin{array}{c} T_{4}={\hat{\Delta }}_{3}^{\text{max}}/S_{2}^{\text{max}}\;\;\text{and}\;\;T_{5}={\hat{\Delta }}_{3}^{\text{max}}/S_{3}^{\text{max}}. \end{array}$$

Alternative standardization procedure of ${\hat{\Delta }}_{1}^{\text{max}}$, ${\hat{\Delta }}_{2}^{\text{max}}$, and ${\hat{\Delta }}_{3}^{\text{max}}$ can also be applied, which is to standardize each component of them and then take the maximum of all standardized components. This alternative standardization procedure leads to the following test statistics for detecting a multivariate two-sample location shift:

$$\begin{array}{c} T_{1}^{*}=\max\left\{\frac{|{\hat{\Delta }}_{11}|}{S_{11}},\frac{|{\hat{\Delta }}_{12}|}{S_{12}},\cdots ,\frac{|{\hat{\Delta }}_{1p}|}{S_{1p}}\right\}, \end{array}$$

$$\begin{array}{c} T_{2}^{*}=\max\left\{\frac{|{\hat{\Delta }}_{21}|}{S_{21}},\frac{|{\hat{\Delta }}_{22}|}{S_{22}},\cdots ,\frac{|{\hat{\Delta }}_{2p}|}{S_{2p}}\right\}, \end{array}$$

$$\begin{array}{c} T_{3}^{*}=\max\left\{\frac{|{\hat{\Delta }}_{21}|}{S_{31}},\frac{|{\hat{\Delta }}_{22}|}{S_{32}},\cdots ,\frac{|{\hat{\Delta }}_{2p}|}{S_{3p}}\right\}, \end{array}$$

$$\begin{array}{c} T_{4}^{*}=\max\left\{\frac{|{\hat{\Delta }}_{31}|}{S_{21}},\frac{|{\hat{\Delta }}_{32}|}{S_{22}},\cdots ,\frac{|{\hat{\Delta }}_{3p}|}{S_{2p}}\right\}, \end{array}$$

and

$$\begin{array}{c} T_{5}^{*}=\max\left\{\frac{|{\hat{\Delta }}_{31}|}{S_{31}},\frac{|{\hat{\Delta }}_{32}|}{S_{32}},\cdots ,\frac{|{\hat{\Delta }}_{3p}|}{S_{3p}}\right\}, \end{array}$$

for *l* = 1, 2, ⋯, 5, in which *S*_*lk*_ denotes the *k*th element of *S*_*l*_, *l* = 1, 2.3. Under the null hypothesis, the proposed test statistics *T*_*l*_ and $T_{l}^{*}$, *l* = 1, 2, ⋯, 5, should be close to zero, whereas they should be far from zero under the alternative hypothesis. When the dimension *p* is equal to 1, these test statistics degenerate to the test statistics introduced by Fried and Dehling [4] and $T_{l}=T_{l}^{*}$ for *l* = 1, 2, ⋯, 5.

### Tests based on *U* statistics

An *U*-statistic recently proposed by Mathur [5] was designated to test a bivariate two-sample location shift. Here, we extend it to serve for detecting the multivariate two-sample location shift. Specifically, the extended *U* test statistic for multivariate sample location detection is defined as

$$\begin{array}{c} U=\sum_{i=1}^{n}\sum_{j=1}^{m}I\left(D_{1i}^{2}>D_{2j}^{2}\right) \end{array}$$ (3)

where $D_{1i}^{2}={∥X_{i}∥}^{2}$ is the Euclidean distance from {*X*_1_, ⋯, *X*_*m*_} to origin and $D_{2j}^{2}={∥Y_{j}∥}^{2}$ is the Euclidean distance from {*Y*_1_, ⋯, *Y*_*n*_} to origin. The null hypothesis is rejected if the observed value of the extended *U* statistic exceeds a critical value of *U* obtained by permutation.

### Implementation: A bootstrap procedure

Here, a bootstrap procedure is introduce to numerically approximate the *p*-values of the proposed robust nonparametric tests. Suppose two random samples with *p*-dimensional independent multivariate observations $X_{1},\cdots ,X_{m}\overset{i.i.d}{∼}F\left(x\right)$ and *p*-dimensional independent multivariate observations $Y_{1},\cdots ,Y_{n}\overset{i.i.d}{∼}G\left(x\right)$ are collected. The null hypothesis *H*_0_: *F*(*x*) = *G*(*x*) and its alternative hypothesis for a location shift in the two multivariate distributions *H*_1_: *F*(*x*) = *G*(*x* + Δ) are considered. To conduct hypothesis testing on such a pair of hypotheses, distributions of the above proposed test statistics are generally unknown in finite samples. Therefore, a bootstrap method can be adopted to approximate the underlying distribution of a test statistic and subsequently determine the corresponding *p*-value. In the bootstrap procedure, a pseudo sample $\left\{X_{i}^{*},i=1,\cdots ,m\right\}$ is drawn from the pooled sample {*X*_*i*_, *Y*_*j*_; *i* = 1, ⋯, *m*, *j* = 1, ⋯, *n*} with replacement, and another pseudo sample $\left\{Y_{j}^{*},j=1,\cdots ,n\right\}$ is drawn from the same pooled sample also with replacement. Let *V* denote any one of the investigated test statistics, and let V* be the bootstrap version of *V* that is calculated from the paired bootstrap pseudo samples $\left\{X_{i}^{*},i=1,\cdots ,m\right\}$ and $\left\{Y_{j}^{*},j=1,\cdots ,n\right\}$. Then, the null hypothesis is rejected if *V* is larger than the (100% ⋅ *α*) quantile of the bootstrap distribution of V*, where *α* is the level of significance of hypothesis testing. It has been confirmed in the literature that the above bootstrap procedure can produce a valid approximation to the test statistic *V* [14–16].

### Implementation: A permutation procedure

A permutation procedure is a competitive alternative to the bootstrap procedure to derive critical values for the proposed robust nonparametric tests. In the permutation procedure, the pooled sample {*X*_*i*_, *Y*_*j*_; *i* = 1, ⋯, *m*, *j* = 1, ⋯, *n*} is repeatedly split to two pseudo samples $\left\{X_{i}^{*},i=1,\cdots ,m\right\}$ and $\left\{Y_{j}^{*},j=1,\cdots ,n\right\}$ without replacement. Let V* denoted the permuted version of a investigated test statistic *V*, and V* is calculated from the paired permuted pseudo samples $\left\{X_{i}^{*},i=1,\cdots ,m\right\}$ and $\left\{Y_{j}^{*},j=1,\cdots ,n\right\}$. Then, the null hypothesis is rejected if *V* is larger than the (100% ⋅ *α*) quantile of the permutation distribution of V*, where *α* is the level of significance of hypothesis testing.

## Simulation studies

This section reports numerical results from a simulation study that was conducted to demonstrate merits of the proposed hypothesis tests and compare them with Hotelling’s *T*^2^. In this simulation study, we aim at examining and comparing performance of the proposed hypothesis tests for detecting a location shift among different pairs of two samples. The sample {*X*_1_, ⋯, *X*_*m*_} was generated from *F*(*x*) and the sample {*Y*_1_, ⋯, *Y*_*n*_} was generated from *G*(*x*). Four different pairs of distributions of *F*(*x*) and *G*(*x*) were considered: (i) *F*(*x*) was a *p*-dimensional multivariate normal distribution *N*_*p*_(1_*p*_, Σ_*p*_), where 1_*p*_ is a *p*-dimensional vector with each component equal to one and Σ_*p*_ is the variance-covariance matrix, and *G*(*x*) was the location shift distribution *N*_*p*_(1_*p*_ + Δ, Σ_*p*_), (ii) *F*(*x*) was a *p*-dimensional multivariate *t* distribution with 1 degrees of freedom *t*_1_(1_*p*_, Σ_*p*_), and *G*(*x*) was the location shift distribution *t*_1_(1_*p*_ + Δ, Σ_*p*_) (iii) *F*(*x*) was a *p*-dimensional multivariate *t* distribution with 3 degrees of freedom *t*_3_(1_*p*_, Σ_*p*_), and *G*(*x*) was the location shift distribution *t*_3_(1_*p*_ + Δ, Σ_*p*_), and (iv) *F*(*x*) was the *p*-dimensional joint distribution of the diagonal elements of a Wishart random matrix that followed the Wishart distribution *W*_*p*_(3, Σ_*p*_), where 3 is the degree of freedom and Σ_*p*_ is the scale matrix, and *G*(*x*) = *F*(*x* + Δ) was the location shift distribution. In this simulation study, two variance-covariance matrices were taken to generated the simulation data: one is an independent variance-covariance matrix *I*_*p*×*p*_, which is a *p* × *p* identify matrix, and another one is a non-independent variance-covariance matrix with diagonal elements equal to 1 and non-diagonal elements equal to 0.5. The dimension *p* of the two samples was set to be 4, and the sample sizes were set as *n* = *m* = 10, 25, and 50. In this simulation study, the location vector Δ in each of four distributions was specified as Δ = (0.5*δ*, *δ*, *δ*, 2*δ*)′, in which *δ* varied to take a value of 0, 0.5, 1, 1.5, or 2. The proposed test statistics ${\hat{\Delta }}_{1}^{\text{max}}$, ${\hat{\Delta }}_{2}^{\text{max}}$, ${\hat{\Delta }}_{3}^{\text{max}}$, *T*_*l*_ and $T_{l}^{*}$, *l* = 1, 2, ⋯, 5, as well as Hotelling’s *T*^2^ and the extended *U* statistic, were applied in two-sample multivariate hypothesis testing to detect the location shift. A total number of 1000 simulation data sets were generated from each pair of specified distributions of the two samples, and then the proposed hypothesis testing was implemented using these investigated test statistics. The rejection rate was subsequently calculated as the frequency that the null hypothesis *H*_0_: *F*(*x*) = *G*(*x*) was rejected among the 1000 simulation data sets by each of the investigated hypothesis test statistics. When *δ* = 0, the pair of true distributions of the two samples have the identical location, and thus the rejection rate is corresponding to simulated Type I error of the hypothesis tests. When *δ* ≠ 0, the pair of true distributions of the two samples reside in different locations, and the rejection rate is corresponding to simulated power of the hypothesis tests. In this simulation study, the number of bootstrap samples was set to be 500 and the significance level was set to be 0.05.

The simulation results of the test statistics based on the bootstrap procedure are presented in S1–S6 Tables. S1–S3 Tables report the Type I errors and power obtained from the simulated paired samples that were generated using the independent variance-covariance matrix with different sample sizes. S4–S6 Tables report the Type I errors and power obtained using the non-independent variance-covariance matrix. It is observed that, when the samples were generated from two multivariate normal distributions with a location shift, Hotelling’s *T*^2^, extended *U* statistic, and *T*_*l*_ and $T_{l}^{*}$, *l* = 2, 3, performed the best among all the investigated test statistics in term of Type I errors and power as *δ* varied. There was not sufficient numerical evidence that the Hotelling’s *T*^2^ statistic outperformed other five statistics. The tests based on the Hodges-Lehmann estimators ${\hat{\Delta }}_{2}^{\text{max}}$, ${\hat{\Delta }}_{3}^{\text{max}}$, *T*_*l*_ and $T_{l}^{*}$, *l* = 2, 3, 4, 5, were more powerful than those based on medians ${\hat{\Delta }}_{1}^{\text{max}}$, *T*_1_ and $T_{1}^{*}$. The choice of the measure of variability within the two samples (i.e., the choice of either *S*_2_ or *S*_3_ and the choice of either $S_{2}^{\text{max}}$ or $S_{3}^{\text{max}}$) had very little impact on the performance of test statistics.

When one sample was generated from a multivariate *t* distribution or a Wishart distribution and another sample was generated from its location shift counterpart, the performance of the proposed robust nonparametric test statistics outperformed the Hotelling’s *T*^2^ and extended *U* statistics in detecting the location shift between the two samples. The power of these robust test statistics was mostly larger than the power given by the Hotelling’s *T*^2^ and extended *U* statistics. Among the nonparametric test statistics, as in the case of multivariate normal distributions, the tests based on the Hodges-Lehmann estimators were more powerful than those based on the medians. The scaled nonparametric tests generally outperform their unscaled counterparts. The nonparametric tests based on *T*_*l*_ and $T_{l}^{*}$, *l* = 2, 3, are most powerful among the investigated test statistics, and the Type I errors given by these four test statistics are mostly close to 0.05. The powers given by the investigated test statistics consistently increased as the location difference between two samples and sample sizes were enlarged.

The simulation results of the test statistics based on the permutation approach are presented in Tables 1–6. Tables 1–3 report the Type I errors and power obtained from the simulated paired samples that were generated using the independent variance-covariance matrix with different sample sizes. Tables 4–6 report the Type I errors and power obtained using the non-independent variance-covariance matrix. It is observed that, when the samples were generated from two multivariate normal distributions with a location shift, the performance of all the test statistics are comparable. Moreover, when one sample was generated from a multivariate *t* distribution or a Wishart distribution and another sample was generated from its location shift counterpart, the performance of the proposed robust nonparametric test statistics outperformed the Hotelling’s *T*^2^ and extended *U* statistics in detecting the location shift between the two samples as it was shown by the bootstrap procedure. The tests based on the Hodges-Lehmann estimators ${\hat{\Delta }}_{2}^{\text{max}}$, ${\hat{\Delta }}_{3}^{\text{max}}$, *T*_*l*_ and $T_{l}^{*}$, *l* = 2, 3, 4, 5, were slightly powerful than those based on medians ${\hat{\Delta }}_{1}^{\text{max}}$, *T*_1_ and $T_{1}^{*}$. A cross comparison of the Type I errors and power given by the bootstrap approach and the permutation approach showed that the permutation approach was able to provide a more stringent control of Type I error and was generally more powerful than the bootstrap procedure. The performance of the nonparametric tests *T*_*l*_ and $T_{l}^{*}$, *l* = 2, 3, 4, 5 did not differ when the permutation approach is applied. Although in Tables 1–6 the scaled nonparametric test statistics cannot be distinguished from their unscaled counterparts, these results were not generalizable since Fried and Dehling [4] had explicitly demonstrated the advantages of the scaled nonparametric test statistics over the unscaled ones.

**Table 1:** Type I errors (*δ* = 0) and power (*δ* ≠ 0) given by the investigated test statistics based on permutation approach in detecting location shift between two samples generated from the four pairs of *F*(*x*) and *G*(*x*) with variance-covariance matrix *I*_4×4_ and sample sizes *n* = *m* = 10.

		Type I errors (*δ* = 0) and power (*δ* ≠ 0)														
*G*(*x*)	*δ* value	*T*^2^	${\hat{\Delta }}_{1}^{\text{max}}$	${\hat{\Delta }}_{2}^{\text{max}}$	${\hat{\Delta }}_{3}^{\text{max}}$	*T*_1_	*T*_2_	*T*_3_	*T*_4_	*T*_5_	$T_{1}^{*}$	$T_{2}^{*}$	$T_{3}^{*}$	$T_{4}^{*}$	$T_{5}^{*}$	*U*
(i)	0	0.040	0.054	0.061	0.058	0.053	0.059	0.057	0.062	0.067	0.056	0.059	0.057	0.058	0.050	0.063
	0.50	0.411	0.353	0.437	0.443	0.334	0.418	0.421	0.433	0.430	0.250	0.358	0.361	0.370	0.371	0.576
	1.00	0.970	0.932	0.977	0.979	0.895	0.977	0.977	0.976	0.977	0.797	0.940	0.941	0.940	0.939	0.993
	1.50	1.000	0.998	1.000	1.000	0.995	1.000	1.000	1.000	1.000	0.994	1.000	1.000	1.000	1.000	1.000
	2.00	1.000	1.000	1.000	1.000	1.000	1.000	1.000	1.000	1.000	1.000	1.000	1.000	1.000	1.000	1.000
(ii)	0	0.018	0.038	0.048	0.048	0.045	0.048	0.050	0.045	0.052	0.038	0.053	0.050	0.049	0.053	0.054
	0.50	0.044	0.118	0.098	0.092	0.136	0.105	0.110	0.094	0.101	0.163	0.143	0.152	0.116	0.125	0.053
	1.00	0.115	0.367	0.286	0.230	0.464	0.325	0.330	0.271	0.273	0.523	0.478	0.501	0.391	0.422	0.087
	1.50	0.233	0.643	0.561	0.438	0.768	0.623	0.617	0.533	0.528	0.813	0.809	0.804	0.717	0.731	0.165
	2.00	0.398	0.802	0.764	0.653	0.922	0.844	0.834	0.770	0.758	0.930	0.938	0.942	0.860	0.881	0.258
(iii)	0	0.026	0.049	0.052	0.054	0.049	0.059	0.056	0.061	0.059	0.048	0.053	0.056	0.060	0.058	0.053
	0.50	0.174	0.241	0.262	0.249	0.240	0.269	0.266	0.264	0.265	0.247	0.273	0.286	0.283	0.279	0.083
	1.00	0.720	0.747	0.810	0.798	0.751	0.818	0.814	0.827	0.813	0.690	0.811	0.823	0.804	0.811	0.364
	1.50	0.945	0.965	0.985	0.979	0.968	0.988	0.988	0.983	0.985	0.953	0.982	0.983	0.984	0.985	0.810
	2.00	0.993	0.997	1.000	1.000	0.995	1.000	1.000	1.000	1.000	1.000	1.000	1.000	1.000	1.000	0.967
(iv)	0	0.023	0.055	0.059	0.047	0.049	0.056	0.063	0.051	0.046	0.047	0.039	0.047	0.041	0.053	0.064
	0.50	0.084	0.101	0.113	0.116	0.087	0.118	0.120	0.118	0.133	0.099	0.114	0.120	0.113	0.117	0.130
	1.00	0.306	0.300	0.334	0.327	0.309	0.351	0.364	0.343	0.360	0.274	0.341	0.379	0.347	0.376	0.345
	1.50	0.652	0.661	0.729	0.667	0.654	0.753	0.759	0.701	0.714	0.589	0.718	0.766	0.689	0.720	0.612
	2.00	0.905	0.904	0.946	0.907	0.897	0.954	0.959	0.933	0.938	0.859	0.966	0.963	0.934	0.939	0.865

**Table 2:** Type I errors (*δ* = 0) and power (*δ* ≠ 0) given by the investigated test statistics based on permutation approach in detecting location shift between two samples generated from the four pairs of *F*(*x*) and *G*(*x*) with variance-covariance matrix *I*_4×4_ and sample sizes *n* = *m* = 20.

		Type I errors (*δ* = 0) and power (*δ* ≠ 0)														
*G*(*x*)	*δ* value	*T*^2^	${\hat{\Delta }}_{1}^{\text{max}}$	${\hat{\Delta }}_{2}^{\text{max}}$	${\hat{\Delta }}_{3}^{\text{max}}$	*T*_1_	*T*_2_	*T*_3_	*T*_4_	*T*_5_	$T_{1}^{*}$	$T_{2}^{*}$	$T_{3}^{*}$	$T_{4}^{*}$	$T_{5}^{*}$	*U*
(i)	0	0.037	0.048	0.047	0.052	0.045	0.049	0.049	0.050	0.052	0.045	0.044	0.040	0.051	0.047	0.049
	0.50	0.847	0.660	0.765	0.770	0.611	0.762	0.768	0.770	0.770	0.557	0.755	0.753	0.744	0.751	0.863
	1.00	1.000	1.000	1.000	1.000	1.000	1.000	1.000	1.000	1.000	0.983	1.000	1.000	1.000	1.000	1.000
	1.50	1.000	1.000	1.000	1.000	1.000	1.000	1.000	1.000	1.000	1.000	1.000	1.000	1.000	1.000	1.000
	2.00	1.000	1.000	1.000	1.000	1.000	1.000	1.000	1.000	1.000	1.000	1.000	1.000	1.000	1.000	1.000
(ii)	0	0.016	0.053	0.067	0.060	0.052	0.062	0.064	0.065	0.064	0.047	0.052	0.054	0.045	0.046	0.049
	0.50	0.032	0.287	0.211	0.179	0.299	0.227	0.223	0.194	0.190	0.365	0.272	0.293	0.235	0.251	0.061
	1.00	0.141	0.829	0.703	0.628	0.867	0.737	0.728	0.674	0.672	0.902	0.816	0.823	0.788	0.793	0.107
	1.50	0.304	0.987	0.960	0.939	0.994	0.974	0.975	0.958	0.958	0.993	0.987	0.990	0.976	0.982	0.267
	2.00	0.485	1.000	0.993	1.000	1.000	1.000	1.000	1.000	1.000	1.000	0.999	0.999	1.000	0.990	0.459
(iii)	0	0.040	0.051	0.051	0.044	0.053	0.052	0.049	0.046	0.041	0.043	0.043	0.046	0.044	0.047	0.048
	0.50	0.432	0.523	0.544	0.545	0.496	0.550	0.548	0.545	0.547	0.494	0.587	0.595	0.579	0.580	0.108
	1.00	0.954	0.989	0.987	0.987	0.976	0.989	0.990	0.990	0.979	0.989	0.991	0.990	0.987	0.991	0.662
	1.50	1.000	1.000	1.000	1.000	1.000	1.000	1.000	1.000	1.000	1.000	1.000	1.000	1.000	1.000	0.984
	2.00	1.000	1.000	1.000	1.000	1.000	1.000	1.000	1.000	1.000	1.000	1.000	1.000	1.000	1.000	1.000
(iv)	0	0.039	0.050	0.056	0.052	0.053	0.055	0.056	0.050	0.050	0.045	0.059	0.053	0.053	0.056	0.070
	0.50	0.179	0.171	0.214	0.181	0.162	0.226	0.232	0.190	0.199	0.148	0.221	0.240	0.195	0.228	0.201
	1.00	0.659	0.606	0.775	0.651	0.576	0.784	0.791	0.684	0.683	0.544	0.764	0.795	0.658	0.701	0.623
	1.50	0.957	0.929	0.984	0.954	0.930	0.989	0.991	0.965	0.968	0.915	0.989	0.991	0.965	0.975	0.912
	2.00	0.998	0.999	0.999	0.995	0.989	1.000	1.000	0.998	0.999	0.984	0.997	0.999	0.996	0.998	0.992

**Table 3:** Type I errors (*δ* = 0) and power (*δ* ≠ 0) given by the investigated test statistics based on permutation approach in detecting location shift between two samples generated from the four pairs of *F*(*x*) and *G*(*x*) with variance-covariance matrix *I*_4×4_ and sample sizes *n* = *m* = 50.

		Type I errors (*δ* = 0) and power (*δ* ≠ 0)														
*G*(*x*)	*δ* value	*T*^2^	${\hat{\Delta }}_{1}^{\text{max}}$	${\hat{\Delta }}_{2}^{\text{max}}$	${\hat{\Delta }}_{3}^{\text{max}}$	*T*_1_	*T*_2_	*T*_3_	*T*_4_	*T*_5_	$T_{1}^{*}$	$T_{2}^{*}$	$T_{3}^{*}$	$T_{4}^{*}$	$T_{5}^{*}$	*U*
(i)	0	0.047	0.054	0.045	0.046	0.055	0.046	0.046	0.046	0.045	0.044	0.048	0.041	0.042	0.045	0.057
	0.50	1.000	0.974	0.999	0.997	0.970	0.999	0.998	0.998	0.999	0.950	0.999	0.998	0.999	0.999	0.999
	1.00	1.000	1.000	1.000	1.000	1.000	1.000	1.000	1.000	1.000	1.000	1.000	1.000	1.000	1.000	1.000
	1.50	1.000	1.000	1.000	1.000	1.000	1.000	1.000	1.000	1.000	1.000	1.000	1.000	1.000	1.000	1.000
	2.00	1.000	1.000	1.000	1.000	1.000	1.000	1.000	1.000	1.000	1.000	1.000	1.000	1.000	1.000	1.000
(ii)	0	0.012	0.051	0.055	0.054	0.049	0.054	0.056	0.055	0.054	0.045	0.054	0.057	0.054	0.050	0.055
	0.50	0.043	0.734	0.589	0.565	0.750	0.602	0.601	0.580	0.571	0.732	0.605	0.604	0.578	0.580	0.126
	1.00	0.171	1.000	0.988	0.983	1.000	0.999	0.999	0.997	0.997	0.999	0.998	0.994	0.997	0.997	0.569
	1.50	0.345	1.000	1.000	1.000	1.000	1.000	1.000	1.000	1.000	1.000	1.000	1.000	1.000	1.000	0.926
	2.00	0.531	1.000	1.000	1.000	1.000	1.000	1.000	1.000	1.000	1.000	1.000	1.000	1.000	1.000	0.996
(iii)	0	0.046	0.052	0.048	0.043	0.052	0.055	0.058	0.052	0.053	0.49	0.048	0.048	0.046	0.045	0.052
	0.50	0.854	0.905	0.921	0.926	0.908	0.923	0.924	0.926	0.927	0.887	0.898	0.919	0.916	0.921	0.226
	1.00	1.000	1.000	1.000	1.000	1.000	1.000	1.000	1.000	1.000	1.000	1.000	1.000	1.000	1.000	0.967
	1.50	1.000	1.000	1.000	1.000	1.000	1.000	1.000	1.000	1.000	1.000	1.000	1.000	1.000	1.000	1.000
	2.00	1.000	1.000	1.000	1.000	1.000	1.000	1.000	1.000	1.000	1.000	1.000	1.000	1.000	1.000	1.000
(iv)	0	0.042	0.047	0.053	0.051	0.052	0.049	0.052	0.051	0.054	0.053	0.047	0.047	0.051	0.050	0.055
	0.50	0.471	0.392	0.591	0.440	0.378	0.589	0.600	0.448	0.461	0.364	0.601	0.620	0.429	0.458	0.376
	1.00	0.982	0.954	1.000	0.967	0.926	1.000	1.000	0.971	0.975	0.930	0.999	0.999	0.977	0.980	0.902
	1.50	1.000	1.000	1.000	1.000	1.000	1.000	1.000	1.000	1.000	1.000	1.000	1.000	1.000	1.000	1.000
	2.00	1.000	1.000	1.000	1.000	1.000	1.000	1.000	1.000	1.000	1.000	1.000	1.000	1.000	1.000	1.000

**Table 4:** Type I errors (*δ* = 0) and power (*δ* ≠ 0) given by the investigated test statistics based on permutation approach in detecting location shift between two samples generated from the four pairs of *F*(*x*) and *G*(*x*) with the non-independent variance-covariance matrix and sample sizes *n* = *m* = 10.

		Type I errors (*δ* = 0) and power (*δ* ≠ 0)														
*G*(*x*)	*δ* value	*T*^2^	${\hat{\Delta }}_{1}^{\text{max}}$	${\hat{\Delta }}_{2}^{\text{max}}$	${\hat{\Delta }}_{3}^{\text{max}}$	*T*_1_	*T*_2_	*T*_3_	*T*_4_	*T*_5_	$T_{1}^{*}$	$T_{2}^{*}$	$T_{3}^{*}$	$T_{4}^{*}$	$T_{5}^{*}$	*U*
(i)	0	0.034	0.056	0.052	0.044	0.052	0.044	0.052	0.038	0.048	0.048	0.046	0.056	0.040	0.050	0.064
	0.50	0.358	0.294	0.364	0.356	0.290	0.332	0.324	0.332	0.328	0.240	0.296	0.298	0.304	0.316	0.178
	1.00	0.974	0.906	0.962	0.964	0.874	0.946	0.944	0.952	0.948	0.752	0.924	0.922	0.922	0.928	0.632
	1.50	1.000	0.995	1.000	0.979	1.000	1.000	1.000	1.000	1.000	0.823	1.000	1.000	1.000	1.000	0.958
	2.00	1.000	0.995	1.000	1.000	0.998	1.000	1.000	1.000	1.000	0.966	1.000	1.000	1.000	1.000	1.000
(ii)	0	0.014	0.044	0.038	0.048	0.044	0.040	0.044	0.044	0.054	0.046	0.044	0.050	0.056	0.040	0.052
	0.5	0.050	0.136	0.104	0.112	0.162	0.112	0.112	0.110	0.114	0.134	0.112	0.134	0.102	0.110	0.072
	1.0	0.252	0.384	0.318	0.276	0.422	0.364	0.384	0.324	0.348	0.452	0.388	0.382	0.346	0.350	0.156
	1.5	0.530	0.742	0.690	0.578	0.824	0.734	0.722	0.670	0.652	0.762	0.744	0.762	0.614	0.652	0.310
	2.0	0.638	0.844	0.814	0.732	0.898	0.862	0.870	0.828	0.804	0.916	0.856	0.870	0.7720	0.788	0.456
(iii)	0	0.024	0.064	0.044	0.040	0.058	0.044	0.044	0.044	0.042	0.046	0.048	0.046	0.042	0.046	0.060
	0.5	0.212	0.252	0.282	0.268	0.238	0.266	0.272	0.264	0.270	0.196	0.218	0.236	0.246	0.254	0.082
	1.0	0.742	0.740	0.784	0.784	0.730	0.780	0.794	0.782	0.782	0.602	0.732	0.750	0.722	0.720	0.312
	1.5	0.958	0.972	0.986	0.984	0.952	0.986	0.986	0.986	0.982	0.900	0.970	0.970	0.966	0.968	0.674
	2.0	0.994	1.000	1.000	1.000	0.992	1.000	1.000	1.000	1.000	0.980	0.998	1.000	0.994	1.000	0.9000
(iv)	0	0.030	0.046	0.047	0.051	0.036	0.051	0.052	0.056	0.053	0.053	0.054	0.048	0.055	0.051	0.066
	0.5	0.069	0.080	0.095	0.096	0.085	0.102	0.102	0.086	0.099	0.085	0.092	0.112	0.096	0.112	0.090
	1.0	0.226	0.262	0.299	0.295	0.251	0.310	0.319	0.311	0.308	0.199	0.293	0.321	0.2780	0.299	0.174
	1.5	0.491	0.583	0.711	0.617	0.582	0.719	0.725	0.651	0.661	0.427	0.625	0.681	0.5470	0.598	0.307
	2.0	0.740	0.851	0.936	0.865	0.835	0.933	0.931	0.885	0.881	0.680	0.870	0.897	0.789	0.850	0.476

**Table 5:** Type I errors (*δ* = 0) and power (*δ* ≠ 0) given by the investigated test statistics based on permutation approach in detecting location shift between two samples generated from the four pairs of *F*(*x*) and *G*(*x*) with the non-independent variance-covariance matrix and sample sizes *n* = *m* = 20.

		Type I errors (*δ* = 0) and power (*δ* ≠ 0)														
*G*(*x*)	*δ* value	*T*^2^	${\hat{\Delta }}_{1}^{\text{max}}$	${\hat{\Delta }}_{2}^{\text{max}}$	${\hat{\Delta }}_{3}^{\text{max}}$	*T*_1_	*T*_2_	*T*_3_	*T*_4_	*T*_5_	$T_{1}^{*}$	$T_{2}^{*}$	$T_{3}^{*}$	$T_{4}^{*}$	$T_{5}^{*}$	*U*
(i)	0	0.033	0.043	0.053	0.059	0.047	0.052	0.058	0.064	0.057	0.046	0.057	0.052	0.062	0.059	0.056
	0.50	0.687	0.629	0.732	0.738	0.608	0.736	0.730	0.731	0.734	0.544	0.703	0.715	0.706	0.715	0.587
	1.00	1.000	0.988	1.000	1.000	0.992	1.000	1.000	1.000	1.000	0.974	1.000	1.000	1.000	1.000	0.994
	1.50	1.000	1.000	1.000	1.000	1.000	1.000	1.000	1.000	1.000	1.000	1.000	1.000	1.000	1.000	1.000
	2.00	1.000	1.000	1.000	1.000	1.000	1.000	1.000	1.000	1.000	1.000	1.000	1.000	1.000	1.000	1.000
(ii)	0	0.011	0.054	0.063	0.065	0.055	0.061	0.060	0.062	0.058	0.063	0.059	0.054	0.063	0.059	0.042
	0.50	0.067	0.291	0.212	0.187	0.320	0.226	0.226	0.208	0.212	0.343	0.244	0.248	0.226	0.232	0.076
	1.00	0.243	0.809	0.693	0.641	0.845	0.736	0.731	0.681	0.681	0.853	0.737	0.737	0.694	0.692	0.273
	1.50	0.446	0.982	0.951	0.918	0.995	0.961	0.955	0.947	0.942	0.991	0.966	0.968	0.947	0.953	0.589
	2.00	0.623	0.998	0.998	0.999	0.998	0.995	0.995	0.994	0.997	0.999	0.997	0.998	0.995	0.997	0.813
(iii)	0	0.028	0.047	0.050	0.057	0.052	0.050	0.051	0.049	0.040	0.045	0.049	0.043	0.054	0.055	0.064
	0.50	0.377	0.515	0.554	0.536	0.516	0.550	0.549	0.544	0.536	0.486	0.547	0.556	0.537	0.542	0.101
	1.00	0.915	0.980	0.988	0.981	0.975	0.992	0.992	0.986	0.988	0.985	0.983	0.984	0.980	0.981	0.658
	1.50	0.995	1.000	1.000	1.000	1.000	1.000	1.000	1.000	1.000	0.997	1.000	1.000	1.000	1.000	0.980
	2.00	0.998	1.000	1.000	1.000	1.000	1.000	1.000	1.000	1.000	1.000	1.000	1.000	1.000	1.000	1.000
(iv)	0	0.040	0.038	0.051	0.054	0.040	0.050	0.055	0.052	0.039	0.054	0.044	0.051	0.056	0.047	0.054
	0.50	0.155	0.150	0.209	0.199	0.167	0.219	0.221	0.198	0.208	0.125	0.209	0.222	0.185	0.211	0.178
	1.00	0.529	0.612	0.771	0.632	0.606	0.788	0.798	0.656	0.682	0.556	0.761	0.791	0.659	0.699	0.573
	1.50	0.874	0.927	0.976	0.925	0.918	0.981	0.983	0.950	0.949	0.911	0.978	0.980	0.934	0.943	0.869
	2.00	0.985	0.994	1.000	0.995	0.998	1.000	1.000	0.998	0.996	0.995	1.000	1.000	0.996	0.995	0.981

**Table 6:** Type I errors (*δ* = 0) and power (*δ* ≠ 0) given by the investigated test statistics based on permutation approach in detecting location shift between two samples generated from the four pairs of *F*(*x*) and *G*(*x*) with the non-independent variance-covariance matrix and sample sizes *n* = *m* = 50.

		Type I errors (*δ* = 0) and power (*δ* ≠ 0)														
*G*(*x*)	*δ* value	*T*^2^	${\hat{\Delta }}_{1}^{\text{max}}$	${\hat{\Delta }}_{2}^{\text{max}}$	${\hat{\Delta }}_{3}^{\text{max}}$	*T*_1_	*T*_2_	*T*_3_	*T*_4_	*T*_5_	$T_{1}^{*}$	$T_{2}^{*}$	$T_{3}^{*}$	$T_{4}^{*}$	$T_{5}^{*}$	*U*
(i)	0	0.036	0.046	0.051	0.053	0.056	0.053	0.053	0.048	0.048	0.052	0.055	0.055	0.047	0.049	0.055
	0.50	0.994	0.955	0.991	0.993	0.993	0.994	0.993	0.992	0.992	0.942	0.991	0.990	0.988	0.987	0.929
	1.00	1.000	1.000	1.000	1.000	1.000	1.000	1.000	1.000	1.000	1.000	1.000	1.000	1.000	1.000	1.000
	1.50	1.000	1.000	1.000	1.000	1.000	1.000	1.000	1.000	1.000	1.000	1.000	1.000	1.000	1.000	1.000
	2.00	1.000	1.000	1.000	1.000	1.000	1.000	1.000	1.000	1.000	1.000	1.000	1.000	1.000	1.000	1.000
(ii)	0	0.011	0.052	0.049	0.046	0.053	0.050	0.049	0.045	0.049	0.049	0.051	0.052	0.049	0.051	0.052
	0.50	0.059	0.734	0.589	0.565	0.752	0.602	0.601	0.583	0.572	0.732	0.603	0.602	0.578	0.580	0.126
	1.00	0.275	1.000	0.992	1.000	1.000	0.996	0.995	0.989	0.990	1.000	0.993	0.992	0.985	0.985	0.569
	1.50	0.500	1.000	1.000	1.000	1.000	1.000	1.000	1.000	1.000	1.000	1.000	1.000	1.000	1.000	0.926
	2.00	0.658	1.000	1.000	1.000	1.000	1.000	1.000	1.000	1.000	1.000	1.000	1.000	1.000	1.000	0.996
(iii)	0	0.037	0.063	0.052	0.053	0.062	0.061	0.050	0.052	0.053	0.057	0.057	0.058	0.057	0.054	0.053
	0.50	0.746	0.905	0.923	0.928	0.908	0.923	0.924	0.926	0.927	0.898	0.919	0.918	0.921	0.920	0.226
	1.00	0.997	1.000	1.000	1.000	1.000	1.000	1.000	1.000	1.000	1.000	1.000	1.000	1.000	1.000	0.967
	1.50	0.999	1.000	1.000	1.000	1.000	1.000	1.000	1.000	1.000	1.000	1.000	1.000	1.000	1.000	1.000
	2.00	1.000	1.000	1.000	1.000	1.000	1.000	1.000	1.000	1.000	1.000	1.000	1.000	1.000	1.000	1.000
(iv)	0	0.038	0.047	0.053	0.054	0.049	0.054	0.051	0.049	0.053	0.052	0.047	0.051	0.047	0.051	0.046
	0.50	0.369	0.392	0.591	0.440	0.378	0.592	0.600	0.448	0.461	0.364	0.601	0.620	0.429	0.458	0.374
	1.00	0.932	0.953	0.992	0.964	0.948	0.996	0.994	0.973	0.977	0.935	0.996	0.995	0.970	0.973	0.902
	1.50	0.999	1.000	1.000	1.000	1.000	1.000	1.000	1.000	1.000	1.000	1.000	1.000	1.000	1.000	1.000
	2.00	1.000	1.000	1.000	1.000	1.000	1.000	1.000	1.000	1.000	1.000	1.000	1.000	1.000	1.000	1.000

Naturally, the proposed nonparametric test statistics function properly without the multivariate normality assumption that the classical Hotelling’s *T*^2^ test requires and therefore are robust to non-normality and outliers. This is the primary reason that we observe in the simulation studies that the proposed tests were comparable to the the Hotelling’s *T*^2^ and the extended *U* tests when the two samples were simulated from multivariate normal distributions and they outperform the two tests when normality does not hold for the simulated samples.

## Statistical analysis of the Thai Healthy Choices study

This section introduces the Thai Healthy Choices study and reports the analysis results from hypothesis testing on effect of a four-session motivational interviewing-based intervention developed in the study to reduce risk behaviors among youth living with HIV [11]. The proposed nonparametric robust test statistics *T*_*l*_ and $T_{l}^{*}$, *l* = 1, 2, ⋯, 5, as well as Hotelling’s *T*^2^ and extended *U* statistic, were applied in the hypothesis testing procedure to detect multivariate difference between intervention and control groups in the study.

### The Thai Healthy Choices study

The Thai Healthy Choices study was conducted at the Thai Red Cross AIDS Research Center in Bangkok [11]. Thai youth living with HIV and attending the Thai Red Cross AIDS Research Centre clinics in Bangkok, who were interested in participation in the study, were referred by their physicians to the study team. The participants eligible to enroll in the study are those between 16 and 25 years old, HIV-positive, and understanding spoken and written Thai enough to participate in study assessments and sessions. Upon completion of consent, participants were randomized in a one-to-one ratio to receive either an designed intervention approach named Healthy Choices (intervention group) or general health education (control group). At the baseline visit, participants completed the assessments. After the baseline visit, participants began to attend either four Healthy Choices sessions in intervention group or four general health education sessions in control group, based on randomization. The sessions in both groups occurred at 1, 2, 6 and 12 weeks after the baseline visit. Each session took approximately 60 min. The assessments similar to the baseline visit were conducted at 1 month after the fourth session and again at 6 months after the fourth session in both groups.

The intervention group received Healthy Choices, a four-session individual-level Motivational Interviewing (MI) counseling that targeted two of three possible risk behaviors, including sexual risks, alcohol use, and antiretroviral adherence. The intervention was delivered in Thai by an MI-trained interventionist. The details of the intervention have been published elsewhere [11]. Session 1 focused on eliciting the participants view of the behavior, exploring barriers as well as sociocultural factors affecting risks and building motivation to initiate the change plan. Session 2 followed a similar format with a focus on the second targeted behaviors. Sessions 3 and 4 were to formalize the personalized behavior change plan, reinforce commitment to change, and identify strategies to maintain healthy behaviors and to prevent relapse. All MI strategies to enhance motivation were used throughout all sessions. The control group received four individualized sessions of general health education unrelated to HIV risk behaviors. Session 1 focused on healthy diet, Session 2 on exercise, and Session 3 on smoking and healthy sleep habits. Session 4 was an overall review of the participants knowledge learned during the prior sessions. The contents of the sessions were adapted from the health education materials published by the Thai Ministry of Public Health. All sessions were delivered didactically by a research assistant who read the contents of the health education manual to the participant. The research assistant received no MI training and was instructed to avoid discussing HIV-related topics, including sexual behavior, HIV disclosure, alcohol and substance use, and medication adherence with the participant.

**Fig 1:**
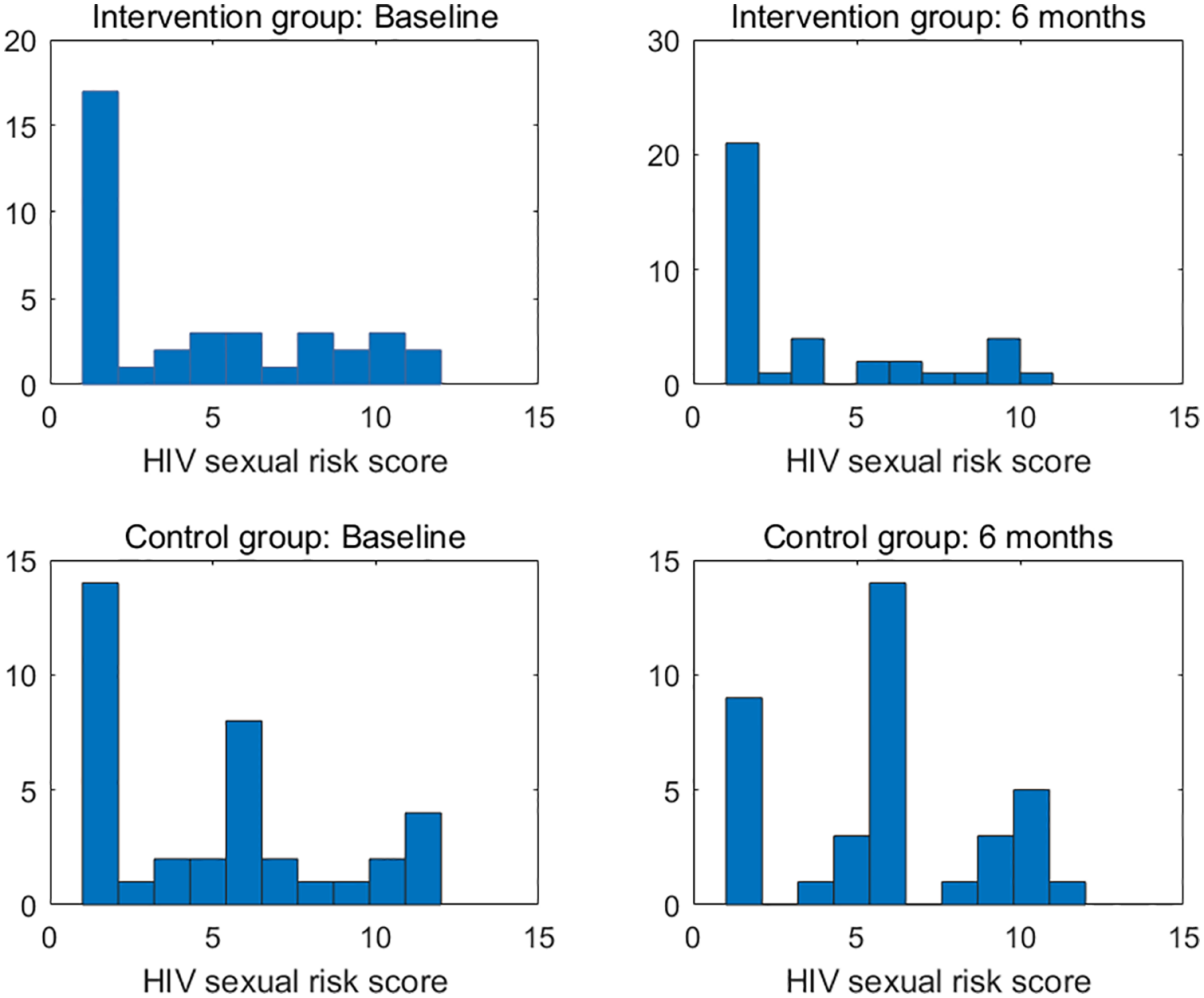
Histograms of HIV sexual risk scores for treatment and control groups at baseline and 6-month visits.

**Fig 2:**
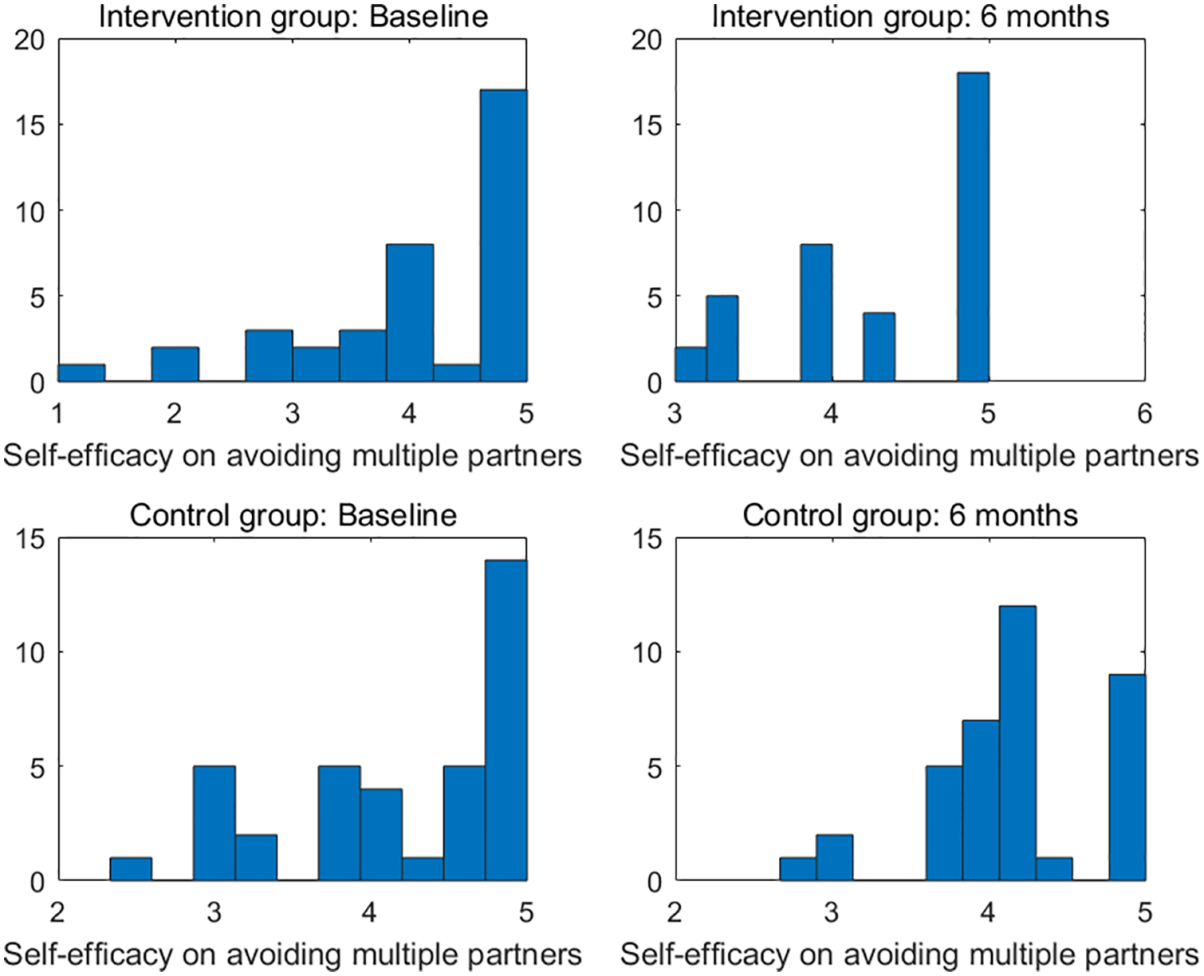
Histograms of self-efficacy on avoiding multiple partners for treatment and control groups at baseline and 6-month visits.

**Fig 3:**
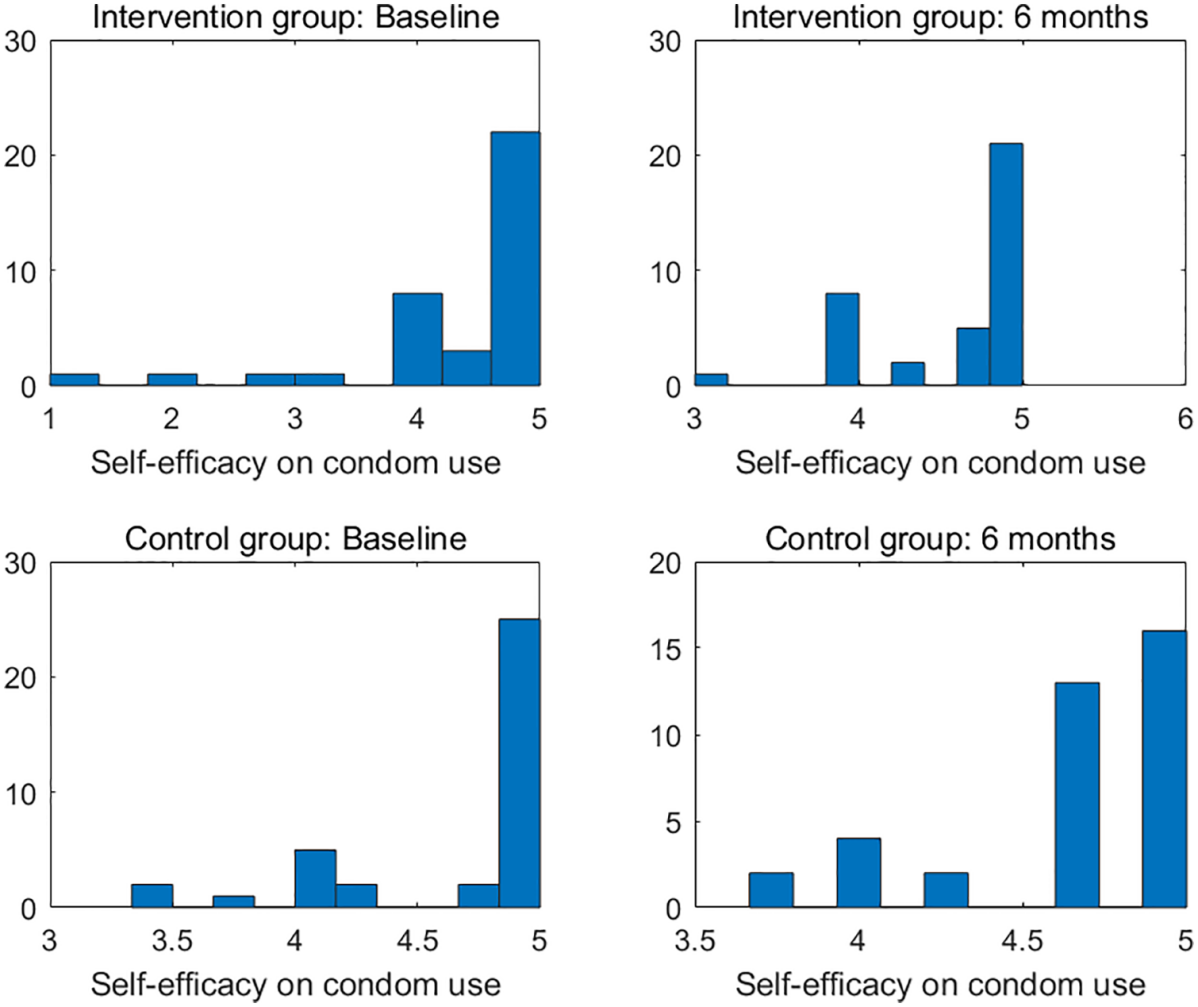
Histograms of self-efficacy on condom use for treatment and control groups at baseline and 6-month visits.

**Fig 4:**
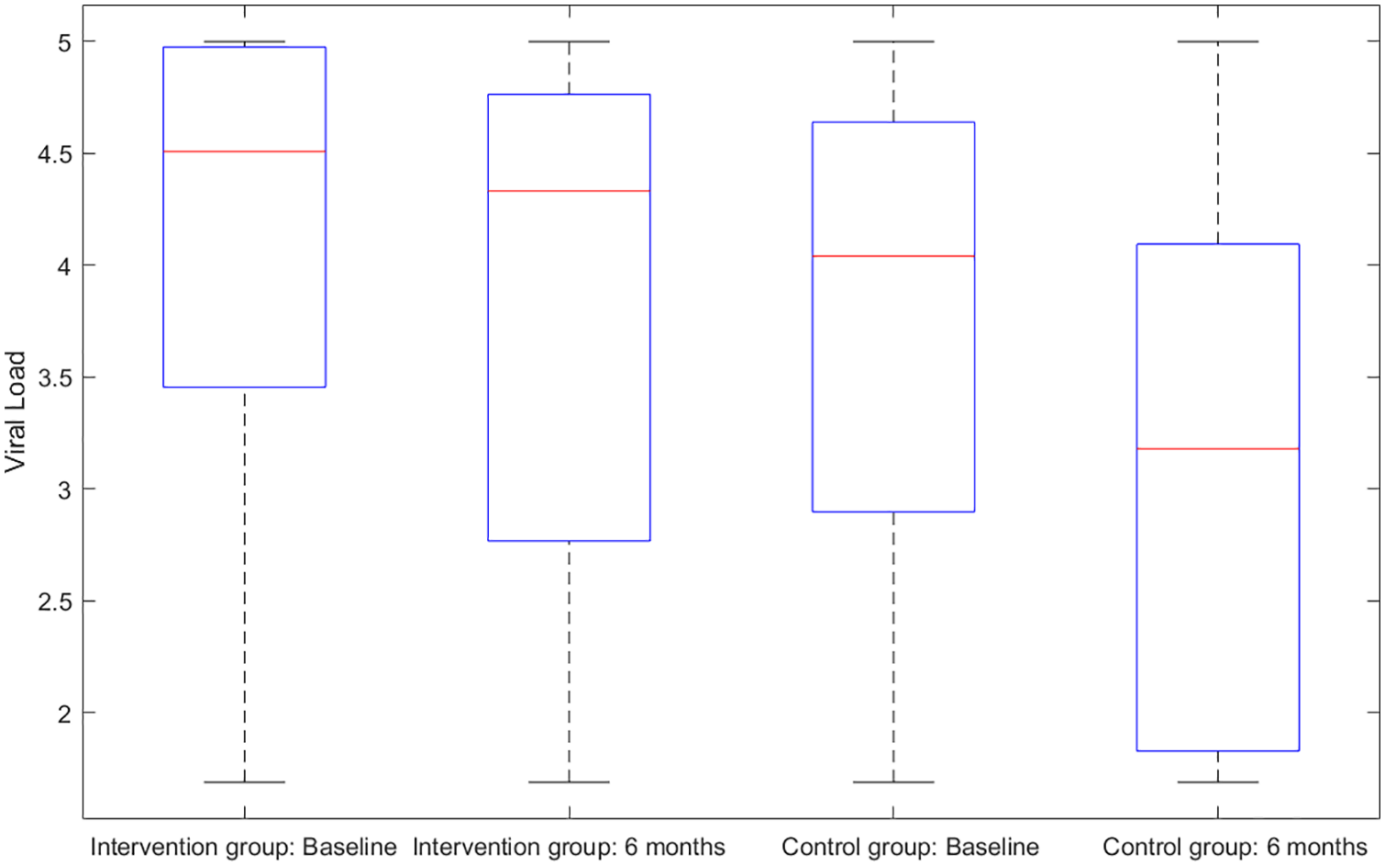
Boxplots of visual load for treatment and control groups at baseline and 6-month visits.

**Fig 5:**
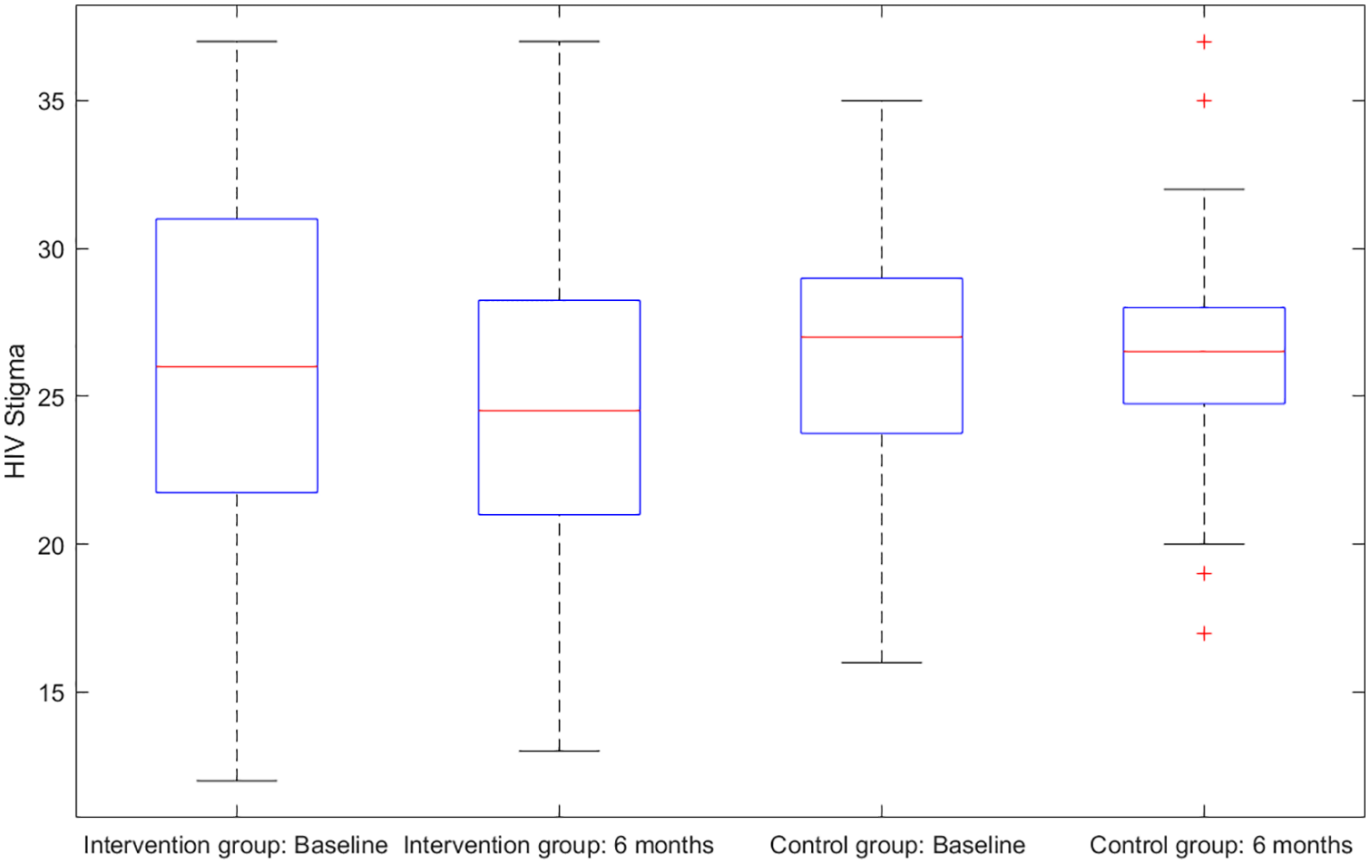
Boxplots of HIV stigma for treatment and control groups at baseline and 6-month visits.

**Fig 6:**
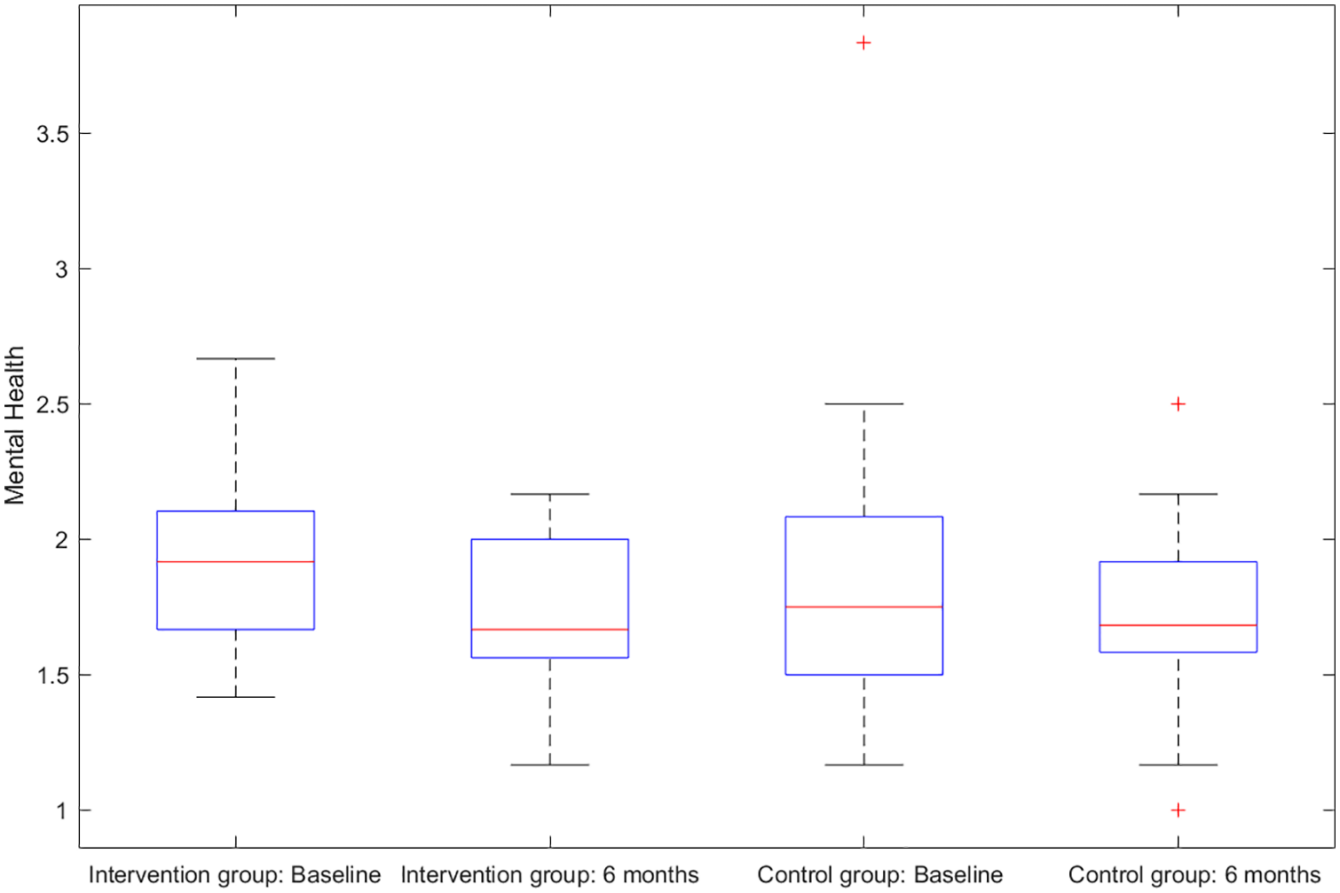
Boxplots of mental health for treatment and control groups at baseline and 6-month visits.

There were six primary clinical measures for the success of the investigated intervention. (1) HIV sexual risk score. An HIV sexual risk scoring system was empirically created based on eight sexual behavior characteristics: sexual intercourse, condom use, number of partners, HIV status of partners, anal sex, receptive anal sex, receptive vaginal sex, and alcohol use with sex. A score (ranging from 1 to 13) was calculated for each participant at each study visit based on the individuals sexual activities in the previous 30 days. The purpose of the scoring system was to provide a broad view of the quantifiable magnitude of an individuals sexual risk. (2) Viral load. Blood samples for plasma HIV viral loads were obtained at baseline, 1 month follow-up, and 6 months follow-up in both study groups and were analyzed by COBAS AmpliPrep/Amplicor HIV-1 Monitor Test, version 1.5 (Roche Molecular Systems, Branchburg, NJ), with the lower limit of detection at 50 copies/ml. (3) HIV stigma. Participants completed the 12-item HIV Stigma Scale, which was developed from Berger’s 40-item HIV Stigma Scale [17]. The measure contains four stigma subscales, with three items per each subscale, representing personalized stigma, disclosure concerns, negative self-image, and public attitude stigma. Cronbach’s *α* was 0.80 in the present study. (4) Mental health. Participants completed the 12-item Thai General Health Questionnaire covering depression, anxiety, social impairment, and somatic complaints. All items were rated on a four-point Likert scale, ranging from 1 (not at all) to 4 (much more than usual). The scores were averaged and a mean score ≥2 was considered clinically significant. Cronbach’s *α* was 0.85 in the present study. (5) Self-efficacy on confidence in avoiding multiple sex partners, and (6) self-efficacy on confidence in using condoms. The Self-Efficacy for Health Promotion and Risk Reduction questionnaire contains 6 items on confidence in using a condom and 3 items on confidence in avoiding sex with multiple partners. Items were rated on a 5-point Likert scale ranging from 1 (very sure I cannot) to 5 (very sure I can). Cronbach’s *α* was 0.89 in the this study. Figs 1–3 display the histograms of HIV sexual risk scores, self-efficacy on avoiding multiple partners, and self-efficacy on condom use for treatment and control groups at baseline and 6-month visits. Figs 4–6 display the boxplots of visual load, HIV stigma, mental health for treatment and control groups at baseline and 6-month visits.

### Hypothesis testing on intervention effect

In the Thai Healthy Choices study, effect of the four-session motivational interviewing-based intervention were simultaneously evaluated by six primary clinical measures: namely HIV sexual risk score, viral load, HIV stigma, mental health, self-efficacy of condom use, and self-efficacy of avoiding multiple sex partners. One approach to determine whether the intervention effect is statistically significant is to conduct a hypothesis test using the nonparametric robust test statistics to determine whether the intervention group and the control group are different in terms of the 6-dimensional multivariate clinical measure at the end of the study (i.e., at 6-month visit). A total number of 74 HIV-positive men who have sex with men were included in this analysis: 37 individuals in intervention group and 37 individuals in control group [18]. Among all participants, 16 of them had missing values and these missing values were replaced with the sample mean of the corresponding variables in each group.

Differences between sample means, medians and two Hodges-Lehmann location estimators of intervention and control groups are reported in S7 Table for each individual clinical measure. These differences demonstrate that the intervention effect may be driven by HIV sexual risk score and HIV stigma. Hypothesis tests were conducted to formally determine whether there was distributional difference between intervention and control groups at baseline and 6-month visits. The null hypothesis was that probability distributions of the multivariate clinical measure for intervention and control groups are identical, and the alternative hypothesis was that there was a location shift between the distributions of the multivariate clinical measure for intervention and control groups. The proposed test statistics *T*_*l*_ and $T_{l}^{*}$, *l* = 2, 3, 4, 5, as well as Hotelling’s *T*^2^ and extended *U* statistic, are applied to detect the location shift. The hypothesis testing results, including the values of test statistics and the corresponding *p*-values, are reported in the upper panel in Table 7. For the baseline samples, all test statistics failed to reject the null hypothesis at the significant level of 0.05, suggesting there was not any statistically significant difference between the probability distributions of the two groups at the baseline visit. For the samples collected at the 6-month follow-up visit, the test statistics *T*_*l*_ and $T_{l}^{*}$, *l* = 2, 3, and Hotelling’s *T*^2^ statistic rejected the null hypothesis but others did not. This implied that distributional locations of the samples collected from the two study groups may be statistically different after 6 months of intervention. Furthermore, we compared the two samples collected at the baseline visit and the 6 month visit within each of the two study groups. The null hypothesis was that probability distributions of the multivariate clinical measure are identical at the baseline and the 6 month visits for the intervention group or for the control group, and the alternative hypothesis was that there was a location shift between the distributions of the multivariate clinical measure at the baseline and the 6 month visits within each group. The hypothesis testing results are reported in the lower panel in Table 7. For the intervention group, the test statistics $T_{2}^{*}$ and $T_{3}^{*}$ rejected the null hypothesis whereas others did not. For the control group, none of the test statistics rejected the null hypothesis.

**Table 7:** Values of test statistics and corresponding *p*-values for comparison of intervention and control groups at baseline and 6-month visits and for comparison of baseline (upper panel) and 6-month visits within each group (lower panel).

		*T*^2^	*T*_2_	*T*_3_	*T*_4_	*T*_5_	$T_{2}^{*}$	$T_{3}^{*}$	$T_{4}^{*}$	$T_{5}^{*}$	*U*
Intervention vs. control at baseline	Statistic value	1.076	0.033	0.033	0.100	0.100	0.500	0.500	1.000	1.000	652
	*p*-value (bootstrap)	0.386	0.788	0.788	0.954	0.952	0.274	0.274	0.288	0.286	0.820
	*p*-value (permutation)	0.386	0.880	0.880	0.982	0.982	0.198	0.246	0.364	0.342	0.768
Intervention vs. control at 6 month	Statistic value	2.747	0.534	0.483	0.443	0.400	0.804	0.689	0.681	0.681	524
	*p*-value (bootstrap)	0.019	0.024	0.048	0.138	0.164	0.040	0.048	0.184	0.184	0.144
	*p*-value (permutation)	0.019	0.024	0.032	0.150	0.172	0.026	0.032	0.144	0.164	0.112
Baseline vs. 6 month for intervention	Statistic value	1.719	0.167	0.167	0.207	0.207	0.667	0.667	0.758	0.758	774
	*p*-value (bootstrap)	0.130	0.578	0.560	0.594	0.590	0.030	0.030	0.386	0.350	0.284
	*p*-value (permutation)	0.130	0.706	0.704	0.552	0.552	0.034	0.026	0.376	0.360	0.292
Baseline vs. 6 month for control	Statistic value	1.420	0.148	0.148	0.173	0.173	0.481	0.514	0.561	0.599	710
	*p*-value (bootstrap)	0.220	0.364	0.360	0.620	0.614	0.230	0.142	0.286	0.270	0.576
	*p*-value (permutation)	0.220	0.418	0.418	0.552	0.552	0.234	0.114	0.220	0.202	0.692

The analysis results of hypothesis testing conclude that there existed statistically significant intervention effect for the four-session motivational interviewing-based intervention developed in the Thai Healthy Choices study to reduce risk behaviors among youth living with HIV. Difference in probability distributions of the multivariate clinical measure for intervention and control groups was detected after 6-month of intervention. Such difference was also confirmed between baseline and 6-month follow-up visits for the intervention group.

## Conclusions

This article proposes a series of robust nonparametric test statistics for detecting location shifts between two multivariate samples. The test statistics are constructed based upon the robust estimators of distribution location, including the medians, the two Hodges-Lehmann estimators, and the extended *U* statistic. Four classes of test statistics are proposed, which include (i) maximum of the component-wise medians or the Hodges-Lehmann estimators, (ii) scaled maximum of the component-wise medians or the Hodges-Lehmann estimators, (iii) maximum of the scaled component-wise medians or the Hodges-Lehmann estimators, and (iv) the extended *U* statistic. The simulation studies suggest that the proposed robust nonparametric test statistics are effective alternatives to the Hotelling’s *T*^2^. The simulation studies also show that the nonparametric tests built upon the Hodges-Lehmann estimators are generally more powerful than others. Numerous nonparametric hypothesis testing procedures have been proposed for comparing a treatment group and a control group in clinical trials with a multivariate endpoint, in the context of nonparametric Behrens-Fisher hypothesis testing problem [19–22]. Further investigation that compares these hypothesis testing procedures with the procedures included in this article may be relevant.

## Supporting information

S1 Table Type I errors (*δ* = 0) and power (*δ* ≠ 0) given by the investigated test statistics based on bootstrap approach in detecting location shift between two samples generated from the four pairs of *F*(*x*) and *G*(*x*) with variance-covariance matrix *I*_4×4_ and sample sizes *n* = *m* = 10. (PDF)

S2 Table Type I errors (*δ* = 0) and power (*δ* ≠ 0) given by the investigated test statistics based on bootstrap approach in detecting location shift between two samples generated from the four pairs of *F*(*x*) and *G*(*x*) with variance-covariance matrix *I*_4×4_ and sample sizes *n* = *m* = 20. (PDF)

S3 Table Type I errors (*δ* = 0) and power (*δ* ≠ 0) given by the investigated test statistics based on bootstrap approach in detecting location shift between two samples generated from the four pairs of *F*(*x*) and *G*(*x*) with variance-covariance matrix *I*_4×4_ and sample sizes *n* = *m* = 50. (PDF)

S4 Table Type I errors (*δ* = 0) and power (*δ* ≠ 0) given by the investigated test statistics based on bootstrap approach in detecting location shift between two samples generated from the four pairs of *F*(*x*) and *G*(*x*) with the non-independent variance-covariance matrix and sample sizes *n* = *m* = 10. (PDF)

S5 Table Type I errors (*δ* = 0) and power (*δ* ≠ 0) given by the investigated test statistics based on bootstrap approach in detecting location shift between two samples generated from the four pairs of *F*(*x*) and *G*(*x*) with the non-independent variance-covariance matrix and sample sizes *n* = *m* = 20. (PDF)

S6 Table Type I errors (*δ* = 0) and power (*δ* ≠ 0) given by the investigated test statistics based on bootstrap approach in detecting location shift between two samples generated from the four pairs of *F*(*x*) and *G*(*x*) with the non-independent variance-covariance matrix and sample sizes *n* = *m* = 50. (PDF)

S7 Table Differences between sample means, medians and two Hodges-Lehmann location estimators of intervention and control groups in baseline and 6-month visits. (PDF)

S1 File Data from the Thai Healthy Choices study. (XLSX)
