# Supplementary material for: Robust multivariate nonparametric tests for detection of two-sample location shift in clinical trials
Source: PLoS One. 2018 Apr 19;13(4):e0195894. doi: 10.1371/journal.pone.0195894 (PMC5908204; doi:10.1371/journal.pone.0195894)
Supplement: S5 Table — (PDF) [file pone.0195894.s005.pdf]

Table S.5: Type I errors ($\delta = 0$) and power ($\delta \neq 0$) given by the investigated test statistics based on bootstrap approach in detecting location shift between two samples generated from the four pairs of $F(x)$ and $G(x)$ with the non-independent variance-covariance matrix and sample sizes $n = m = 20$.

		Type I errors ($\delta = 0$) and power ($\delta \neq 0$)														
$G(x)$	δ value	T^2	$\hat{\Delta}_1^{\max}$	$\hat{\Delta}_2^{\max}$	$\hat{\Delta}_3^{\max}$	T_1	T_2	T_3	T_4	T_5	T_1^*	T_2^*	T_3^*	T_4^*	T_5^*	U
(i)	0	0.033	0.017	0.033	0.030	0.037	0.042	0.043	0.032	0.039	0.023	0.037	0.037	0.027	0.029	0.053
	0.50	0.687	0.452	0.743	0.725	0.474	0.753	0.754	0.741	0.743	0.434	0.725	0.733	0.703	0.711	0.527
	1.00	1.000	0.988	1.000	1.000	0.992	1.000	1.000	1.000	1.000	0.974	1.000	1.000	1.000	1.000	0.994
	1.50	1.000	1.000	1.000	1.000	1.000	1.000	1.000	1.000	1.000	1.000	1.000	1.000	1.000	1.000	1.000
	2.00	1.000	1.000	1.000	1.000	1.000	1.000	1.000	1.000	1.000	1.000	1.000	1.000	1.000	1.000	1.000
(ii)	0	0.011	0.025	0.035	0.010	0.041	0.046	0.050	0.023	0.025	0.030	0.041	0.044	0.018	0.020	0.058
	0.50	0.067	0.096	0.143	0.069	0.230	0.216	0.226	0.123	0.140	0.210	0.206	0.218	0.126	0.132	0.066
	1.00	0.243	0.614	0.603	0.410	0.815	0.716	0.716	0.561	0.581	0.786	0.711	0.722	0.564	0.584	0.233
	1.50	0.446	0.909	0.929	0.772	0.985	0.951	0.955	0.885	0.886	0.981	0.960	0.963	0.891	0.903	0.545
	2.00	0.623	0.990	0.986	0.926	0.998	0.995	0.995	0.974	0.977	0.999	0.997	0.998	0.972	0.977	0.807
(iii)	0	0.028	0.010	0.037	0.027	0.022	0.045	0.051	0.039	0.040	0.024	0.039	0.043	0.034	0.035	0.059
	0.50	0.377	0.326	0.491	0.456	0.416	0.558	0.550	0.516	0.516	0.376	0.516	0.523	0.478	0.487	0.086
	1.00	0.915	0.941	0.988	0.981	0.964	0.992	0.992	0.986	0.988	0.935	0.983	0.984	0.980	0.981	0.580
	1.50	0.995	1.000	1.000	1.000	1.000	1.000	1.000	1.000	1.000	0.997	1.000	1.000	1.000	1.000	0.966
	2.00	0.998	1.000	1.000	1.000	1.000	1.000	1.000	1.000	1.000	1.000	1.000	1.000	1.000	1.000	1.000
(iv)	0	0.040	0.012	0.040	0.027	0.027	0.050	0.055	0.032	0.039	0.024	0.044	0.051	0.026	0.027	0.054
	0.50	0.155	0.076	0.181	0.117	0.110	0.209	0.224	0.152	0.164	0.105	0.194	0.224	0.125	0.154	0.128
	1.00	0.529	0.403	0.721	0.520	0.470	0.775	0.792	0.599	0.636	0.415	0.733	0.768	0.563	0.630	0.495
	1.50	0.874	0.819	0.976	0.905	0.867	0.981	0.983	0.929	0.933	0.814	0.972	0.977	0.912	0.928	0.840
	2.00	0.985	0.973	1.000	0.995	0.984	1.000	1.000	0.998	0.996	0.975	1.000	1.000	0.996	0.995	0.975
