# Supplementary material for: Robust multivariate nonparametric tests for detection of two-sample location shift in clinical trials
Source: PLoS One. 2018 Apr 19;13(4):e0195894. doi: 10.1371/journal.pone.0195894 (PMC5908204; doi:10.1371/journal.pone.0195894)
Supplement: S7 Table — (PDF) [file pone.0195894.s007.pdf]

Table S.7: Differences between sample means, medians and two Hodges-Lehmann location estimators of intervention and control groups in baseline and 6-month visits.

		Risk Score	Viral Load	HIV Stigma	Mental Health	Multiple Partners	Condom Use
Baseline	Mean	−0.432	0.252	−0.568	0.099	−0.126	−0.279
	Median	−1.000	0.468	−1.000	0.167	−0.667	−0.333
	$\hat{\Delta}_2$	0.000	0.121	0.000	0.167	0.000	0.000
	$\hat{\Delta}_3$	−0.500	0.307	−0.500	0.125	−0.167	−0.333
6 month	Mean	−1.912	0.619	−2.005	0.044	0.211	−0.020
	Median	−4.500	1.152	−2.005	−0.017	0.211	0.333
	$\hat{\Delta}_2$	−2.412	0.692	−2.000	0.044	0.211	0.000
	$\hat{\Delta}_3$	−2.000	0.797	−1.762	0.044	0.327	0.000
